# Transperineal ultrasound-guided prostate biopsy is safe even when patients are on combination antiplatelet and/or anticoagulation therapy

**DOI:** 10.1186/s12894-017-0245-z

**Published:** 2017-07-05

**Authors:** Kimitoshi Saito, Satoshi Washino, Yuhki Nakamura, Tsuzumi Konishi, Masashi Ohshima, Yoshiaki Arai, Tomoaki Miyagawa

**Affiliations:** 1 Department of Urology, Jichi Medical University Saitama Medical Center, 1-847, Amanuma-cho, Omiya-ku, Saitama, 330-8503 Japan; 2 Department of Urology, Nishi-Omiya Hospital, 1-1173, Mihashi, Omiya-ku, Saitama, 330-0856 Japan

**Keywords:** Anticoagulant, Antiplatelet, Complication, Dual antiplatelet therapy, Transperineal prostate biopsy

## Abstract

**Background:**

To assess whether hemorrhagic complications associated with transperineal prostate biopsy increased in patients on antiplatelet and/or anticoagulant therapy.

**Methods:**

In total, 598 consecutive patients underwent transperineal prostate biopsy. The medication group comprised patients who took anti-thromboembolic agents, and the control group comprised those who did not take these agents. No anti-thromboembolic agent was stopped before, during, or after prostate biopsy in the medication group. Complications developing in both groups were compared and classified using the modified Clavien classification system. Subgroup analyses to compare complications in patients taking single antiplatelet, single anticoagulant, and dual antiplatelet and/or anticoagulant agents, and multivariate analyses to predict bleeding risk were also performed.

**Results:**

Of the 598 eligible patients, 149 comprised the medication group and 449 comprised the control group. Hematuria (Grade I) developed in 88 (59.1%) and 236 (52.5%) patients in the medication and control group, respectively (*p* = 0.18). Clot retention (Grade I) was more frequently observed in the medication group than the controls (2.0% versus 0.2%, respectively, *p* < 0.05). Hospitalization was more frequently prolonged in the medication than the control group (4.0% versus 0.4% of patients, respectively). No complication of Grade III or higher developed in either group. Hematuria was more frequent in patients taking a single anticoagulant (*p* = 0.007) or two anti-thromboembolic agents (*p* = 0.04) compared with those taking a single antiplatelet agent. Other complications were generally similar among the groups. In the multivariate analysis, taking more than two anti-thromboembolic agents was the only significant risk factor for bleeding events.

**Conclusion:**

No severe complication developed after the transperineal biopsies in either group, although minor bleeding was somewhat more frequent in the medication group. It may not be necessary to discontinue anticoagulant and/or antiplatelet agents when transperineal prostate biopsy is contemplated.

## Background

Systematic prostate biopsy is routinely performed on patients with suspected prostate cancer, to obtain tissue cores [1]. Certain hemorrhagic complications including hematuria, hematospermia, and rectal and perineal bleeding, have been reported [2]. Recently, urologists are increasingly encountering patients with multiple comorbidities including coronary arterial disease that earlier required percutaneous coronary arterial intervention with angioplasty, together with placement of bare metal or drug-eluting stent (DES). Other patients have cardiac dysrhythmias such as atrial fibrillation, valvular heart disease, or deep vein thrombosis. In others, inferior vena cava filters have been placed [3]. These comorbidities are managed using an increasing array of oral antiplatelet (AP) and anticoagulant (AC) drugs; such patients require comprehensive management to mitigate the risk of complications after urological interventions. DESs are often placed in patients with coronary artery disease; such patients cannot simply stop taking AP drugs in the perioperative period surrounding prostate biopsy.

When patients taking such drugs require surgical intervention, including transrectal ultrasound (TRUS) -guided prostate biopsy, one of the following three medication options must be chosen after detailed discussion between doctor and patient and the giving of informed consent: drug continuation, interruption, or use of a heparin bridge. Several studies have shown that aspirin increases the incidence of minor but not severe bleeding events during TRUS-guided biopsy [4–7]. An International Consultation on Urological Disease/American Urological Association (ICUD/AUA) review reported that prostate biopsy is safe for patients taking low-dose aspirin; the risk of minor bleeding is only approximately one-third higher than in controls [3]. However, these studies featured transrectal, not transperineal biopsy. Furthermore, it has been but seldom studied whether patients on AC therapy, dual antiplatelet therapy (DAPT), or combination AP and AC therapy, can safely undergo prostate biopsy while remaining on these drugs.

This study evaluated whether transperineal prostate biopsy could be performed safely on patients taking Aps or ACs, without discontinuing the drugs.

## Methods

### Patients

This was a retrospective observational study approved by our local institutional review board. A total of 598 eligible patients underwent prostate biopsy (with collection of more than 14 tissue cores from each), on suspicion of prostate cancer, at Jichi Medical University Saitama Medical Center, from July 2011 to December 2015. The AP/AC group comprised patients taking APs or ACs, and the remainder constituted the control group. The APs were aspirin, cilostazol, clopidogrel, and ticlopidine. The ACs were warfarin, rivaroxaban, dabigatran, and apixaban. No AP or AC drug was stopped before, during, or after the prostate biopsy.

### Biopsy protocol

All of the biopsies were performed via the transperineal approach, using an 18-gauge needle (Tru-Core II Automatic Biopsy Instrument, ARGON MEDICAL DEVICES, Athens, TX, USA) under general or spinal anesthesia. Each TRUS-guided systematic biopsy typically yielded 14 cores, eight from the peripheral lobe and six from the transitional lobe. In patients with suspicious lesions evident on magnetic resonance imaging (MRI), two additional targeted cores were collected for each lesion. All targeted biopsies were performed using a cognitive registration technique, as described previously, with some modification [8]. As perioperative antimicrobial prophylaxis, levofloxacin 500 mg was prescribed once daily for 3 days. The patients were discharged the next morning.

### Evaluation of comorbidities

All of the patients were evaluated about 2 weeks after the prostate biopsy at the first outpatient visit. They were asked about the incidence of bleeding-associated events and other complications. Bleeding-associated events were gross hematuria, perineal hematoma, and clot retention. Events observed at least once were included. Gross hematuria was defined as passing blood or red urine. Perineal hematoma was defined as a non-pulsatile mass > 1.5 cm in diameter diagnosed by visual examination and palpation of the perineum. Clot retention was defined as acute urinary retention due to clot. Hematospermia was not evaluated. Other complications were acute urinary retention and infection. Prolonged hospitalization was defined as a patient who was not discharged the next morning. Comorbidities were classified using the modified Clavien classification system [9].

### Statistical analysis

The types and proportions of complications between the AP/AC and control groups were compared. Subgroup analysis was performed among groups taking single APs, single ACs, and dual APs and/or ACs. Statistical analysis was performed with the aid of GraphPad Prism ver. 5.0 or SPSS ver. 19.0. Data were compared using Student’s *t*-test, the Mann–Whitney *U*-test, or the chi-square test. Multivariate analysis was performed using logistic regression analysis to determine significant predictors of bleeding events. The hazard ratio and 95% confidence interval were determined. All data shown are means and standard deviations, or medians with interquartile ranges (IQR). Statistical significance was set at *p* < 0.05.

## Results

### Patient characteristics and biopsy outcomes

A total of 598 eligible patients underwent prostate biopsies: 149 took APs or ACs (AP/AC group), while 449 did not take APs or ACs (control group). Table 1 shows the patient characteristics and biopsy outcomes. Patients in the AP/AC group were significantly older than those in the control group (71.6 ± 6.7 vs. 68.7 ± 7.5 years; *p* < 0.0001). The prostate-specific antigen (PSA) level, prostate volume, number of biopsy cores collected, and cancer detection rate did not significantly differ between the two groups.

**Table 1:** Patient characteristics and cancer detection rate

	Control, *n* = 449	AP/AC, *n* = 149	
	mean (SD)	mean (SD)	*p* value
Age	68.7 (7.5)	71.6 (6.7)	< 0.0001
PSA, ng/mL	10.3 (8.5)	11.4 (15.4)	0.44
Prostate volume, cm^3^	50.2 (24.3)	51.1 (27.8)	0.75
Biopsy cores, median (range)	16 (14–22)	16 (14–22)	0.67
Cancer detection, *n* (%)	254 (56.6)	93 (62.4)	0.21

*PSA* Prostate specific antigen, *AP* Antiplatelet, *AC* Anticoagulant

### AP and AC therapy

Of the 149 patients in the AP/AC group, 80, 34, 19, and 16 were on single AP, single AC, DAPT, and combination AP and AC therapy, respectively (Table 2). Most patients in the single AP and single AC subgroups were taking aspirin (65/80 patients) or warfarin (25/34 patients). The daily dosage of aspirin, cilostazol, clopidogrel, and ticlopidine was 100 mg, 75 mg, 100–200 mg, and 100–200 mg, respectively. The daily dosage of warfarin, rivaroxaban, dabigatran, and apixaban was 0.56–4.75 mg, 10–15 mg, 150–300 mg, and 5 mg, respectively. The median (IQR) prothrombin time- international normalized ratio in patients taking warfarin was 1.735 (1.420–2.053).

**Table 2:** Details of the antiplatelet and anticoagulant therapies

		*n* = 149	(%)
Single	Antiplatelet		
	Aspirin	65	(43.6)
	Cilostazol	7	(4.7)
	Clopidogrel	5	(3.4)
	Ticlopidine	3	(2.0)
	Total	80	(53.7)
	Anticoagulant		
	Warfarin	25	(16.8)
	Rivaroxaban	4	(2.7)
	Dabigatran	4	(2.7)
	Apixaban	1	(0.7)
	Total	34	(22.8)
Dual	Dual antiplatelet therapy		
	Aspirin + Clopidogrel	14	(9.4)
	Aspirin + Ticlopidine	3	(2.0)
	Aspirin + Cilostazol	2	(1.3)
	Total	19	(12.8)
	Antiplatelet + Anticoagulant		
	Aspirin + Warfarin	10	(6.7)
	Other combinations	6	(4.0)
	Total	16	(10.7)

### Reasons for taking APs or ACs

Table 3 shows the reasons for taking APs or ACs. Ischemic heart disease, atrial fibrillation, and cerebral infarction were the first (34.9%), second (30.9%), and third (17.4%) most common reasons.

**Table 3:** Reason for taking antiplatelet or anticoagulant therapy

	*n* = 149	(%)
Ischemic heart disease	52	(34.9)
Atrial fibrillation	46	(30.9)
Cerebral infarction	26	(17.4)
Heart valve replacement surgery	13	(8.7)
Arteriosclerosis obliterans or cervical arterial stenosis	8	(5.4)
Primary prophylaxis	11	(7.4)
Others/Unknown	6	(4.1)

### *Complication*s *in the AP and/or AC group* versus *the control group*

Complications after prostate biopsy in the two groups are shown in Table 4. Grade I complications: the frequency of hematuria was similar in both groups (59.1% in the AP/AC vs. 52.5% in the control group; *p* = 0.18); three patients (2.0%) of the AP/AC group experienced clot retention, the incidence of which was significantly higher than in the control group (one [0.2%] patient; *p* = 0.049); two (1.3%) and three patients (0.7%) of the AP/AC and control groups, respectively, experienced perineal hematoma (*p* = 0.60); four (2.7%) and seven (1.6%) patients in the AP/AC and control groups, respectively, experienced acute urinary retention (*p* = 0.48). Grade II complications: two (1.3%) and one (0.2%) patient in the AP/AC and control groups, respectively, experienced urinary tract infection (*p* = 0.15). No Grade III or higher complication developed in either group. Six AP/AC patients required prolonged hospitalization, the incidence of which was significantly higher than in the control group (two patients [0.4%]; *p* = 0.01). The causes of prolonged hospitalization in AP/AC group patients were clot retention in three, perineal hematoma in one, acute urinary retention in one, and aspiration pneumonitis in one; both control group patients had perineal hematomas. No patient of either group required re-admission.

**Table 4:** Complications in the antiplatelet/anticoagulant group versus the control group

Modified Clavien-Dindo Classification	Complications	Control *n* = 449	AP/AC *n* = 149	
		*n* (%)	*n* (%)	*p* value
I	Hematuria	236 (52.5)	88 (59.1)	0.18
	Perineal hematoma	3 (0.7)	2 (1.3)	0.60
	Acute urinary retention	7 (1.6)	4 (2.7)	0.48
	Clot retention	1 (0.2)	3 (2.0)	< 0.05
II	Urinary tract infection	1 (0.2)	2 (1.3)	0.15
	Prolonged hospitalization	2 (0.4)	6 (4.0)	< 0.05

*AP* Antiplatelet, *AC* Anticoagulant

### Complications in the single AP vs. the single AC vs. the dual therapy subgroups

The complications among subgroups taking single APs, single ACs, and dual antithrombotic agents are compared in Table 5. Hematuria was more frequent in the single AC (*p* = 0.007) and dual antithrombotic subgroups (*p* = 0.04) than the single AP subgroup. No other complication, nor the need for prolonged hospitalization, differed significantly among subgroups.

**Table 5:** Complications in the single antiplatelet or single anticoagulant versus the dual therapy subgroups

Modified Clavien Classification	Complications	Single, *n* = 114		Dual, *n* = 35
		AP, *n* = 80 *n* (%)	AC, *n* = 34 *n* (%)	*n* (%)
I	Hematuria	38 (47.5)	26 (76.5)*	24 (68.6)*
	Perineal hematoma	0	2 (5.9)	0
	Acute urinary retention	1 (1.3)	2 (5.9)	1 (2.9)
	Clot retention	3 (3.8)	0	0
II	Urinary tract infection	0	0	2 (5.7)
	Prolonged hospitalization	4 (5.0)	1 (2.9)	1 (2.9)

* *p* < 0.05 vs. AP group, *AP* Antiplatelet, *AC* Anticoagulant

### Risk factors for bleeding events

We performed a multivariate analysis to predict which patients were more likely to have bleeding events by comparing patients who experienced bleeding events, such as hematuria, perineal hematoma, and clot retention (*n* = 302), with those who did not (*n* = 257). Patients who had missing data were excluded from this analysis (*n* = 39). The results are shown in Table 6. Taking more than two APs/ACs was a significant predictor of bleeding events (Hazard ratio [95% CI]: 2.230 [1.028–4.839], *p* = 0.042), while age, PSA levels, number of biopsy cores, and prostate volume were not significant.

**Table 6:** Multivariate analysis to predict the risk factors for bleeding events

	Variable	N	Hazard ratio	95% CI	*p* value
Age	< 70	294	-	-	
	≥ 70	265	0.931	0.657–1.319	0.687
PSA, ng/ml	< 6.0	158	-	-	
	6.0–9.9	214	0.839	0.551–1.277	0.413
	≥ 10	187	0.696	0.450–1.076	0.103
Number of biopsy cores	< 16	204	-	-	
	≥ 16	355	0.974	0.683–1.389	0.883
Prostate volume, cm^3^	< 45.0	278	-	-	
	≥ 45.0	281	1.135	0.805–1.600	0.470
No. of AP/AC agents	0	420	-	-	
	1	105	1.339	0.863–2.076	0.193
	≥ 2	34	2.230	1.028–4.839	0.042

*PSA* Prostate specific antigen, *AP* Antiplatelet, *AC* Anticoagulant, *CI* Confidence interval

## Discussion

In the study, we found the following that complications arising after transperineal biopsy were generally similar in patients who were or were not taking AP/AC, although clot retention was somewhat more frequent in patients taking AP/AC agents (Table 4). In addition, patients who were taking more than two AP/AC agents were more likely to experience bleeding events (Table 6), and no complication of grade III or higher were noted in any group.

It has been suggested that 10–12 systematic biopsy cores are required for a histopathological diagnosis of prostate cancer [1]. Recently, targeted biopsies of suspicious MRI lesions have improved prostate cancer detection. However, biopsy of not only targeted lesions but also systematic prostate biopsy is required [10–12]. As the number of biopsy cores increases, possible hemorrhagic complications must be considered.

In this study, we examined the safety of transperineal prostate biopsy in patients taking APs/ACs compared with controls (not on APs or ACs). The frequency of hematuria was similar in the AP/AC and control groups (Table 4). However, hemorrhagic events requiring treatment, such as clot retention and transperineal hematoma, were somewhat more frequent in the AP/AC than the control group, which might increase the frequency of prolonged hospitalization in the AP/AC group.

Halliwell et al. reported that 72% of patients taking aspirin experienced hematuria after transrectal biopsy; this value was significantly higher than the control value of 62% [5]. By contrast, Chowdhury et al. found that hematuria developed after transrectal biopsy in 28, 34, and 37% of patients on warfarin, aspirin, and controls, respectively; these proportions did not significantly differ [6]. The meta-analysis of Carmignani et al. [13] showed that hematuria was significantly more frequent among patients taking aspirin than controls (odds ratio [OR] of 1.36). However, the increased risk was for minor bleeding only. The ICUD/AUA have stated that prostate biopsy can be performed safely on patients on low-dose aspirin; the risk of minor bleeding is only approximately one-third higher than in the controls [3]. However, this suggestion held only for transrectal, and not transperineal, biopsy. Recently, Asano et al. [14] studied whether it was safe to continue antithrombotic agents in patients undergoing transperineal biopsy and found that the frequencies of hemorrhagic complications after transperineal biopsy were similar among control, continued drug administration, and interrupted drug administration groups; no severe hemorrhagic complications occurred, consistent with our results.

Patients on DAPT are increasing in line with the popularization of DESs in patients with coronary artery disease. Other patients take AP/AC combinations to treat various conditions. However, it has been unclear whether prostate biopsy was safe when patients were on two antithrombotics. Recently, Raheem et al. found that patients taking APs and/or ACs (5 and 11% of all patients studied were on aspirin-plus-warfarin or aspirin-plus-clopidogrel, respectively) had fewer hematuria episodes than controls [15]. We found that the frequency of complications did not generally differ among subgroups taking single APs, single ACs, or dual anti-thromboembolic agents, although hematuria was more frequent in the single AC and dual anti-thromboembolic subgroups than in the single AP subgroup (Table 5). In the multivariate analysis, the number of AP/AC agents was the only significant predictor of bleeding events (Table 6). No complication of grade III or higher was encountered in any group. Therefore, patients who are taking two APs or ACs may experience minor bleeding events more frequently, but will not experience severe complications after transperineal prostate biopsy.

Aspirin non-adherence/withdrawal is associated with a three-fold higher risk of major adverse cardiac events (OR 3.14) [16]. This risk is magnified in patients with intracoronary stents; discontinuation of AP treatment was associated with an even higher risk of adverse events (OR 89.78). Furthermore, aspirin interruption increases the incidence of ischemic stroke/transient ischemic attack (OR 3.4) [17].

In 493 patients who stopped continuous anticoagulation therapy to allow the performance of dental procedures, 5 (1%) developed severe embolic complications, including four deaths [18]. Discontinuation of ACs might increase the risk of severe embolic complications. When warfarin is discontinued, a heparin bridge is recommended [19]. However, heparin bridging is controversial because it was recently demonstrated that a heparin bridge did not decrease the risks of ischemic stroke or systemic embolism in stable non-valvular atrial fibrillation [20]. Overall, discontinuation of APs/ACs is associated with severe thrombotic events; thus, the continuation of these drugs is ideal. When patients taking anti-thromboembolic agents are scheduled to undergo transperineal TRUS-guided prostate biopsy, clinicians and patients can select one of two following plans: to continue these agents with the risk of minor bleeding or to interrupt these agents with the risk of rare severe thromboembolic complications. We believe that it is more reasonable to continue anti-thromboembolic agents than to refuse to accept the slight risk of a mild bleeding event.

There were several limitations in this study. First, the retrospective nature of the work increased the risk of patient selection bias. Second, we did not assess the extent or duration of hematuria; thus, we do not know if they differed among groups. However, no Grade III or higher complication developed in any group.

## Conclusions

No Grade III or higher complication developed after transperineal biopsy in either the medication or control group, although clot retention was observed somewhat more frequently in the medication group. It may not be necessary to discontinue anticoagulant or antiplatelet agents when transperineal prostate biopsy is performed.

## Abbreviations

AC: Anticoagulant
AP: Antiplatelet
AUA: American Urological Association
DAPT: Dual antiplatelet therapy
DES: Drug-eluting stent
ICUD: International Consultation on Urological Disease
IQR: Interquartile range
MRI: Magnetic resonance imaging
OR: Odds ratio
PSA: Prostate specific antigen
TRUS: Transrectal ultrasound
